# Recurrent prescription of sleep medication among primary care patients with type 2 diabetes: an observational study of real-world registry data

**DOI:** 10.1186/s12875-023-02045-1

**Published:** 2023-04-05

**Authors:** Eveliina Heikkala, Jari Jokelainen, Ilona Mikkola, Juha Auvinen, Maria Hagnäs

**Affiliations:** 1 Rovaniemi Health Center, Koskikatu 25, Rovaniemi, 96200 Finland; 2 Research Unit of Population Health, University of Oulu, PO Box 5000, Oulu, 90015 Finland; 3 Medical Research Center Oulu, University of Oulu and Oulu University Hospital, PO Box 5000, Oulu, 90014 Finland; 4 Arctic Biobank, Infrastructure for Population Studies, Faculty of Medicine, Northern Finland Birth Cohorts, University of Oulu, PO Box 5000, Oulu, 90015 Finland

**Keywords:** Type 2 diabetes, Primary care, Sleep medication

## Abstract

**Background:**

Little knowledge exists on the prevalence of recurrent sleep medication prescriptions among primary care patients with type 2 diabetes (T2D). Our aims were to examine the prevalence of recurrent sleep medication prescriptions and to elucidate the most often prescribed sleep medications in a Finnish primary care T2D population.

**Methods:**

The study examined 4,508 T2D patients who consulted a primary health care center between 2011 and 2019 in Rovaniemi, Finland. All the data were retrieved from patient records, and recurrent sleep medication was defined as two or more prescriptions within the study period. We used the Chi-square and Kruskal–Wallis tests to compare patients who did and did not have recurrent sleep medication prescriptions.

**Results:**

Altogether 28.1% of the T2D patients had been prescribed recurrent sleep medication. Benzodiazepine-like medication, melatonin, and mirtazapine were most often prescribed (to 56.9%, 44.4%, and 35.8%, respectively). Only 22.0% of the patients with recurrent sleep medication prescriptions had been diagnosed with a sleep disorder.

**Conclusions:**

Recurrent sleep medication prescriptions are frequent among primary care T2D patients. It seems that sleep disorders are underdiagnosed in relation to this. Primary care clinicians should carefully estimate the need for sleep medication when treating T2D patients’ sleep problems and emphasize the diagnostic patterns of sleep problems.

**Supplementary Information:**

The online version contains supplementary material available at 10.1186/s12875-023-02045-1.

## Background

Diagnostic-level sleep disorders affect between 5% and 20% of the general population in Western countries [1, 2]. Despite current guidelines that recommend non-medication-based treatment methods for long-term sleep disorder [1], the rate and use of prescribed sleep medication is frequent, especially among older populations [3–7]. Patients with type 2 diabetes (T2D) are an important patient group in this regard, as they are more likely than patients without T2D to suffer from sleep problems [8, 9].

As is well acknowledged, long-term exposure to sleep medication is associated with pervasive adverse health outcomes, including an increased risk of falls and fractures [10], cognitive impairment [11], and suicidal attempts [12]. It also often leads to dependence [13], which is one reason why the majority of patients report ceasing long-term sleep medication to be difficult [5]. With respect to T2D, melatonin [14, 15], which is commonly used sleep medication, may also play a role in glucose metabolism, although no clear consensus on this has been reached. Moreover, the sleep problems behind prescribed sleep medication tend to confuse conclusions; that is, sleep problems have a negative impact on glucose balance [16], whereas improved sleep due to treatment may result in better outcomes [8].

A recent cohort study estimated that around 16% of all T2D patients have been prescribed recurrent sleep medication [9]. However, less data exist on the prevalence of recurrent sleep medication prescriptions in a primary care setting. This knowledge would be highly valuable in the design of T2D treatment guidelines targeted at primary care clinicians. Moreover, there may be cultural differences in the prescription tendency of sleep medications, so guidelines on recommended medication are not necessarily fully comparable [1, 17, 18].

Therefore, we sought to primarily explore (a) what is the prevalence of recurrent sleep medication prescriptions among Finnish primary care T2D patients and (b) which are the most common medications prescribed. As a secondary aim, we studied whether the T2D patients who had been prescribed recurrent sleep medication (divided into three subcategories: previous, ongoing, subsequent medication), differed from those who had not been prescribed sleep medication in terms of sleep disorder diagnosis and several background and treatment target variables, including the achievement of glycosylated hemoglobin A1c (HbA1c), low-density lipoprotein (LDL), and systolic blood pressure (sBP) goals. We hypothesized that recurrent sleep medication prescriptions are common, and that melatonin is the most often prescribed medication.

## Methods

### Study sample and ethics

**Fig. 1:**
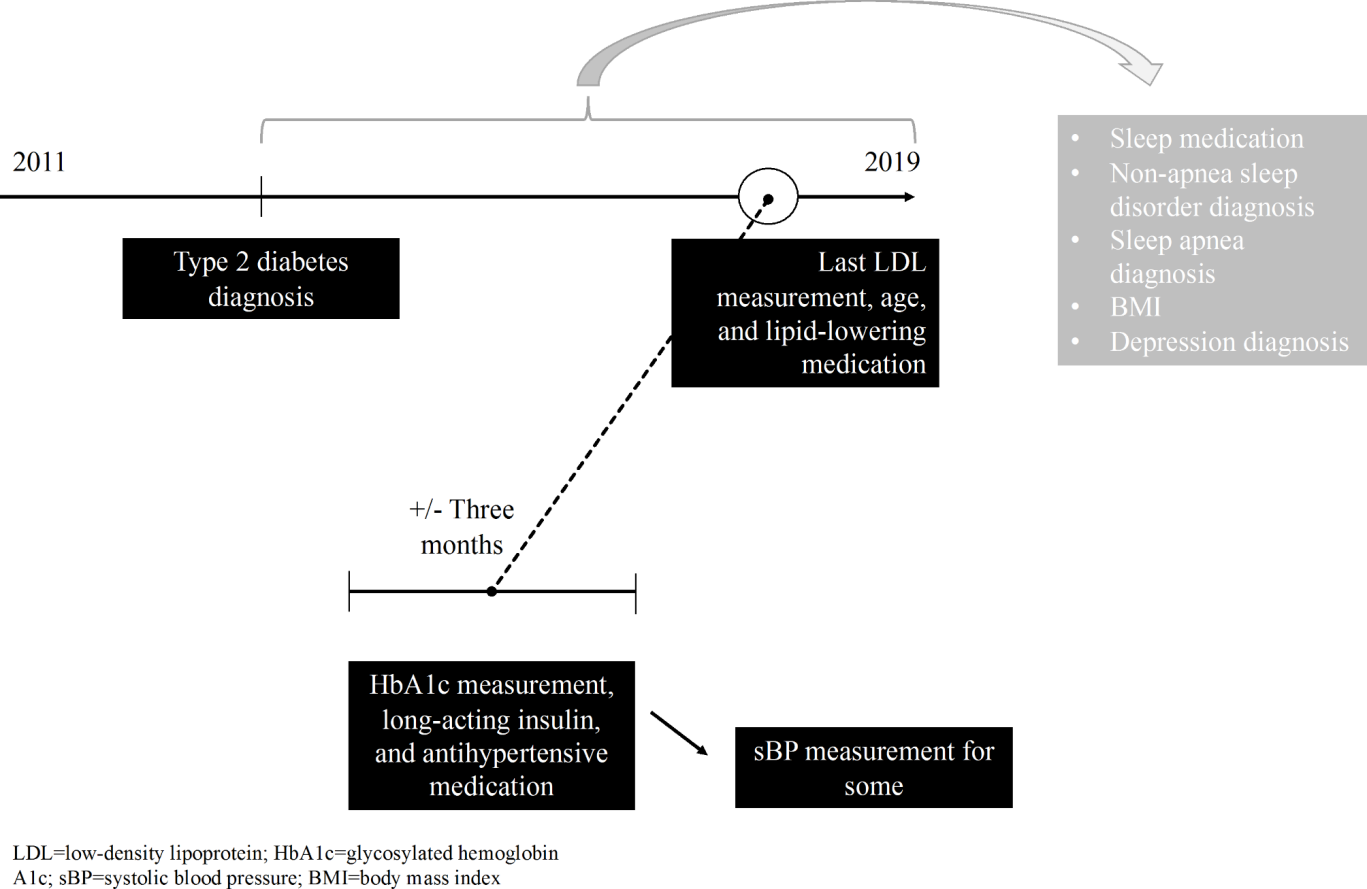
Schematic model of data collection (presented for a hypothetical patient). Type 2 diabetes could have been diagnosed at any point between 2011 and 2019, and all other included data were collected after that diagnosis (data on treatment target variables [LDL, HbA1c, sBP] were connected to a certain measurement point, while other data were not)

This study was part of the Rovaniemi Primary Care T2D Study. The study population was drawn from patients who had been diagnosed T2D and who attended a primary health care center in Rovaniemi, Finland between 2011 and 2019 (‘study period’) (n = 5,104). T2D diagnosis was based on the 10th revision of the World Health Organization’s International Classification of Disease (ICD-10) codes E11.1–E11.9 and code T90 of the International Classification of Primary Care (ICPC). Patients who had not contacted the health care center at least twice within a minimum of one year during the study period were excluded (n = 308) on the basis of the fundamental idea that all the included patients had had the option of more than one sleep medication prescription within the study period. With respect to the HbA1c, LDL, and sBP measurements, we first obtained the latest LDL value after T2D diagnosis from the registers (Fig. 1). Then, HbA1c had to have been measured three months before or after the LDL measurement. If this was not the case, the patients were excluded from the study. Finally, we gathered the home-monitored sBP measurements (a mean of four double measurements) obtained within the six-month periods, from the registers, but did not use the availability of this information as an exclusion criteria. Measurements related to weight and height were taken during the control appointments at the health care center if a patient returned a blank preliminary information form. All assessed variables were collected from patient records after T2D diagnosis was recorded (Fig. 1).

The final study sample was drawn from T2D patients who had sleep medication prescription, LDL, and HbA1c data available after T2D diagnosis (n = 4,508; Fig. 1). Due to its registry-based design, no written consent was required for this study according to the national legislation. The study was approved by the Ethics Committee of the Lapland Central Hospital, Rovaniemi, Finland (Reg. no.05/2018). Data were pseudonymized and handled at group-level only.

### Sleep medication and sleep medication categories

The medication data were based on ATCs (Anatomical Therapeutic Codes) and T2D diagnosis was required to precede these data. The included medications were: temazepam (ATC: N05CD07), benzodiazepine-like medication (N05CF), melatonin (N05CH01), mirtazapine (N06AX11; only prescriptions for 15 mg pills were considered), nitrazepam (N05CD02), doxylamine (R06AA09), doxepin (N06AA12; only prescriptions for 3 mg pills were considered), trimipramine (N06AA10; only prescriptions for 25 mg pills were considered), and trazodone (N06AX05; only prescriptions for 50 mg pills were considered). These were selected on the basis of the national guideline [18]. We decided to exclude oxazepam and diazepam prescriptions as we had no data on the indication for which these medications were prescribed; that is, both can also be prescribed for anxiety [19].

As we were interested in recurrent prescriptions of sleep medication (yes/no), we first identified those who had been prescribed at least two sleep medications within the study period and those who had been prescribed no sleep medication or had only one prescription during the study period. We labeled these patient groups “recurrent sleep medication prescription” and “no recurrent sleep medication prescription” respectively [9]. Then, we linked these groups to measurement points of treatment target measurements (see further information above). On the basis of the consecutive connection between sleep medication prescription and the HbA1c, LDL, and sBP measurement points, the “recurrent sleep medication prescription” category patients were further divided into three subcategories: 1) previous medication (over a year before HbA1c, LDL, and sBP measurements), 2) ongoing medication (within a year before the measurements), and 3) subsequent medication (after the measurements). If patients were eligible to belong to two or more subcategories, the ongoing medication subcategory was prioritized, followed by the previous medication and subsequent medication subcategories.

### Background variables and treatment targets of T2D

Sex (female/male), age (a continuous variable), non-apnea sleep disorders (any of the following ICD-10 codes: F51.x or G47.x [excluding G47.3 = sleep apnea]; yes/no), sleep apnea (ICD-10 code G47.3; yes/no), body mass index (BMI; a continuous variable), depression diagnosis (any of the following ICD-10 codes: F32, F33, F34.1, F41.2; yes/no), achievement of treatment targets for HbA1c (< 53 mmol/mol vs. 53 or over), LDL (< 2.5 mmol/l vs. 2.5 or over), and sBP (< 135 mmHg vs. 135 or over), prescription of long-acting insulin (any of the following ATC codes: A10AE, A10AC, and A10AD; yes/no), antihypertensive medication of any kind (any of the following ATC codes: C09A, C09C, C07AB, C08CA, and C03; yes/no), and lipid-lowering medication of any kind (any of the following ATC codes: C10AA and C10AX; yes/no) were retrieved from patient records after the T2D diagnosis. BMI was presented as weight divided by height squared (kg/m^2^). For the analyses, we calculated the mean of all BMI measurements after the T2D diagnosis for each patient. The treatment target variables (HbA1c, LDL, and sBP) were dichotomized on the basis of the national guidelines [20].

### Statistical analysis

The numbers and percentages, mean and standard deviations (SDs), or median and interquartile range were calculated for categorical and continuous variables, respectively. The Chi-square or Kruskal–Wallis test was used in the analyses to compare the background and treatment target variables of the patients who had been prescribed recurrent sleep medication (dichotomous and four-category variables) and of those who had not. The level of statistical significance was set at p = 0.05. All statistical analyses were conducted using R, R Studio.

## Results

The final study sample consisted of 4,508 T2D patients who had data on sleep medication, and for whom HbA1c and LDL target achievements were available. Their average age was 69.9 years (SD: 11.9) and 45.8% were females (Table 1). Sleep apnea was diagnosed among 12.5% and a non-apnea sleep disorder among 12.2% of all the patients. A total of 70.7% and 54.9% had achieved the treatment targets of HbA1c and LDL, respectively, whereas 46.1% (n = 620/1,346) had attained the sBP goal.

**Table 1:** Characteristics of study sample, stratified by recurrent sleep medication prescription. Percentages and numbers presented and full data available, unless otherwise indicated

	Recurrent sleep medication prescription (n = 1,266)	No recurrent sleep medication prescription (n = 3,242)	Total (n = 4,508)	P value
Age, mean (SD)	73.4 (11.8)	68.5 (11.7)	69.9 (11.9)	< 0.001
Sex				< 0.001
Females	53.9 (683)	42.6 (1,381)	45.8 (2,064)	
Males	46.1 (583)	57.4 (1,861)	54.2 (2,444)	
Sleep medication (percentage of ‘yes’ responses are presented)				
Benzodiazepine-like medication	56.9 (720)	1.0 (33)	16.7 (753)	< 0.001
Melatonin	44.4 (562)	0.9 (28)	13.1 (590)	< 0.001
Mirtazapine	35.8 (453)	0.5 (17)	10.4 (470)	< 0.001
Temazepam	19.2 (243)	0.3 (11)	5.6 (254)	< 0.001
Nitrazepam	0.2 (3)	0 (0)	0.1 (3)	0.006
Doxylamine	0.5 (6)	0 (0)	0.1 (6)	< 0.001
Doxepin	0 (0)	0 (0)	0 (0)	
Trimipramine	5.4 (68)	0.1 (4)	1.6 (72)	< 0.001
Trazodone	0 (0)	0 (0)	0 (0)	
Number of sleep medications, median (IQR)	10.0 (4.0–25.0)	NA		
Time in months between sleep medication prescriptions, median (IQR)	2.94 (1.19–6.17)	NA		
Sleep apnea diagnosis				< 0.001
Yes	23.4 (296)	8.3 (268)	12.5 (564)	
No	76.6 (970)	91.7 (2,974)	87.5 (3,944)	
Non-apnea sleep disorder diagnosis				< 0.001
Yes	22.0 (278)	8.3 (270)	12.2 (548)	
No	78.0 (988)	91.7 (2,972)	87.8 (3,960)	
Body mass index, mean (SD)	29.3 (6.3)	30.0 (5.8)	29.8 (6.0)	< 0.001
*Missing*	n = 231	n = 784	n = 1,015	
Depression diagnosis				< 0.001
Yes	20.1 (255)	7.2 (234)	10.8 (489)	
No	79.9 (1,011)	92.8 (3,008)	89.2 (4,019)	
Long-acting insulin				< 0.001
Yes	32.1 (406)	21.8 (707)	24.7 (1,113)	
No	67.9 (860)	78.2 (2,535)	75.3 (3,395)	
Antihypertensive medication of any kind				< 0.001
Yes	85.6 (1,084)	78.5 (2,544)	80.5 (3,628)	
No	14.4 (182)	21,5 (698)	19.5 (880)	
Lipid-lowering medication of any kind				0.219
Yes	67.9 (860)	66.0 (2,140)	66.5 (3,000)	
No	32.1 (406)	34.0 (1,102)	33.5 (1,508)	
Achieved HbA1c target (< 53 mmol/mol)				< 0.001
Yes	66.4 (841)	72.4 (2,348)	70.7 (3,189)	
No	33.6 (425)	27.6 (894)	29.3 (1,319)	
Achieved LDL target (< 2.5 mmol/l)				< 0.001
Yes	59.2 (750)	53.2 (1,724)	54.9 (2,474)	
No	40.8 (516)	46.8 (1,518)	45.1 (2,034)	
Achieved sBP target (< 135 mmHg)				0.398
Yes	44.7 (243)	47.0 (377)	46.1 (620)	
No	55.3 (301)	53.0 (425)	53.9 (726)	
*Missing*	n = 722	n = 2,440	n = 3,162	

HbA1c = glycosylated hemoglobin A1c

LDL = low-density lipoprotein

sBP = systolic blood pressure

SD = standard deviation

IQR = interquartile range

NA = not applicable

A total 28.1% of the T2D patients had been prescribed recurrent sleep medication within the study period (Table 1). The most often prescribed medications were benzodiazepine-like medications (56.9%), followed by melatonin (44.4%) and mirtazapine (35.8%). The patients who had been prescribed recurrent sleep medication were slightly older than those who had not (73.4 [SD 11.8] vs. 68.5 [SD11.7], p < 0.001), were more likely to be female (53.9% vs. 42.6%, p < 0.001), and had more likely been diagnosed with depression (20.1% vs. 7.2%, p < 0.001) (Table 1). Only 22.0% of these patients had been diagnosed with a non-apnea sleep disorder.

In terms of subcategories of recurrent sleep medication (Supplement 1), patients who had ongoing sleep medication were more likely to be female (55.8%) and had more likely been diagnosed with sleep apnea (24.8%) and depression (22.7%), compared with the other subcategories and ‘no recurrent sleep medication prescription’ category (p < 0.001 for all). The HbA1c target was most likely to be achieved by the ‘no recurrent sleep medication prescription’ category patients (72.4%). In turn, the LDL target was most likely to be attained by the ‘recurrent sleep medication prescription’ category patients (59.2%) (Table 1), and particularly by patients who had previously been prescribed recurrent sleep medication (63.3%) (Supplement 1). There were no significant differences between the two main sleep medication categories in terms of achieving their sBP target (Table 1), but over half the patients in the ‘subsequent medication’ subcategory had achieved the goal (54.3%), compared less than a half in other subcategories and in the ‘no recurrent sleep medication prescription’ category (Supplement 1).

## Discussion

In extensive, real-world data on 4,508 T2D patients, we found that 28.1% had been prescribed recurrent sleep medication during the study period between 2011 and 2019. The most commonly prescribed medications were benzodiazepine-like medication (56.9%), melatonin (44.4%), and mirtazapine (35.8%). Interestingly, a non-apnea sleep disorder was only diagnosed among 22.0% of the T2D patients with recurrent prescriptions. None of the sleep medication (sub-)categories clearly outperformed in all treatment balance measurements.

In our data, nearly one-third of the T2D patients had been prescribed sleep medication at least twice. This finding is particularly alarming as the most often prescribed medications were benzodiazepine-like medications which are related to adverse health effects [10, 12] and hence, only recommended for short-term use [18]. Whether sedative sleeping drugs (such as benzodiazepine-like medication) should be entirely avoided, particularly among older patients [21], which the present study sample also included, is under debate. Unfortunately, it seems that older patients aged 65 or over have long-term prescriptions for sedative sleep medication five to six times more often than younger ones [22, 23].

As for the present prescription estimates, it is worth noting that the prevalence of recurrent sleep medication users may be even higher than can be indirectly estimated from the records, as the availability of melatonin products is not regulated in Finland and they are also available as self-care drugs. There are no other Finnish studies on recurrent sleep medication prescriptions among T2D patients, but one Scandinavian study has detected a clearly lower prevalence rate of recurrent prescriptions than that of ours when evaluating prescriptions of benzodiazepine-like medication and melatonin in a T2D sample [9]. This difference may be related to the older study sample (70 years on average vs. median age 63 years) or a higher number of medications being included in the present study (nine vs. two).

Still, the extent to which the treatment of sleep problems relies on medications and on non-pharmacological methods should be explored. Based on comparisons to other reports, we can speculate that medication may play a crucial part in the treatment of sleep problems among primary care T2D patients. According to the current European guidelines [1], non-medication-based treatment methods, such as cognitive behavioral therapy, should be first-line treatment for sleep problems, and this should also be the case in terms of T2D patients. On the other hand, the prescription prevalence of sleep medication may reflect a substantially high frequency of sleep problems in this population even though the sleep disorder diagnoses themselves were at lower levels; only slightly over one-fourth of patients with recurrent sleep medication prescriptions had been diagnosed with a non-apnea sleep disorder. In this sense, a sleep disorder diagnosis may not represent the magnitude of sleep problems in real life. A similar phenomenon was observed in a previous registry-based study of the Danish population, in which only 1% of all patients with a sleep disorder were identified on the basis of their diagnoses, and 99% on the basis of recurrent sleep medication [9]. These observations raise questions about whether primary care physicians follow the recommended procedures of the diagnostic management of sleep disorders [1]. Although a limited number of non-apnea sleep disorders in relation to the prescription prevalence may be related to under-recording, i.e., diagnoses not being appropriately recorded in patient records, it is possible that sleep disorders are not properly diagnosed among T2D patients in the primary care context.

Contrary to expectations, patients with ongoing sleep medication only slightly differed from those without recurrent sleep medication prescriptions in terms of achieving their HbAc1, LDL, and sBP goals; that is, they were less likely to fulfill their HbA1c target, but more likely to attain their LDL target. The discrepancy in the HbA1c goal may be due to the observation that a higher percentage of patients in the ‘ongoing medication’ subcategory than in the ‘no recurrent sleep medication prescription’ category had been prescribed long-acting insulin, which is indicative of worse T2D. As discussed above, systematic reviews and meta-analyses repeatedly demonstrate that sleep medication prescriptions likely reflect sleep problems related to higher glucose levels among T2D patients [24, 25]. In contrast, reports relating sleep problems to cholesterol are more conflicting [24, 26, 27], with a recent meta-analysis showing no such association [24]. A slightly higher prevalence of lipid-lowering prescriptions in the ‘ongoing medication’ subcategory than in the ‘no recurrent sleep medication prescription’ category may indicate that more of the former use lipid-lowering medication, which could at least partly explain the LDL finding. In future, a case-control study in which patients’ age, gender, BMI, and severity of diabetes are controlled for, or a randomized control trial would serve as better grounds for clarifying the role of recurrent ongoing sleep medication in the T2D treatment balance in more detail.

Sleep apnea has been recognized as a highly common comorbidity among T2D patients, with the highest estimates being up to 90% for overall prevalence and 59% for moderate-to-severe sleep apnea [28, 29]. Therefore, the finding that sleep apnea was only diagnosed among 12.5% of our study population was surprising. On the other hand, this is in line with the literature that shows that sleep apnea is highly underdiagnosed among primary care T2D patients [28]. In their extensive primary care study, Heffner et al., [28] found that 18% of T2D patients had a sleep apnea diagnosis, which is close to our estimates, but far from those expected. Primary care physicians should pay more attention to symptoms of sleep apnea and screen them regularly when treating T2D patients [30], particularly due to the increased morbidity and mortality risks related to these co-existing diseases [31].

Its substantial registry-based data is the main strength of this T2D study. This is also the first study on recurrent sleep medication prescriptions among the Finnish primary care T2D population. In terms of sex distribution and average age [32], the study sample was similar to that from which T2D data was collected in several primary care clinics throughout Finland. This increases the generalizability of the current results to Finnish primary care patients with T2D. However, several elements need to be regarded as limitations in this study. First, the design was cross-sectional and thus no cause-and-effect conclusions can be drawn. Secondly, the study sample included mainly older patients, which may limit the generalizability of the current results to working-aged samples. On the other hand, a substantial part of primary care T2D patients are older in Finland because employed individuals are treated by occupational health services. Thus, we believe that the study sample likely represents the Finnish primary care population of T2D patients quite well in this regard. Thirdly, due to the structure of our available registers, we had no data on the reasons for which the medications were prescribed, thus some of them may have been prescribed for reasons other than sleep problems. Moreover, we lacked information on whether the patients had actually taken the drugs, which can be seen as a limitation and restricts conclusions on sleep medication prescriptions only.

## Conclusions

Based on the current results drawn from 4,508 primary care patients with T2D, recurrent sleep medication prescriptions are frequent and benzodiazepine-like medications are the most often prescribed drugs. These findings do not reflect the current guidelines of sleep disorders. It seems that sleep disorders are underdiagnosed in relation to recurrent sleep medication prescription. Primary care clinicians should carefully estimate the need for sleep medication when treating T2D patients’ sleep problems, and emphasize the diagnostic pattern of sleep problems. Moreover, sleep apnea should be screened more effectively among T2D patients. In future, the ongoing treatment strategies of sleep disorders within the T2D populations should be evaluated in detail.

## Electronic supplementary material

Below is the link to the electronic supplementary material.

Supplementary Material 1

## Data Availability

The data that support the findings of this study are available from Rovaniemi City Health Services, but restrictions apply to the availability of these data, which were used under license for the current study, and so are not publicly available. Data are however available from the corresponding author, Maria Hagnäs upon reasonable request and with permission of Rovaniemi City Health Services.

## Abbreviations

ATC: Anatomical Therapeutic Code
BMI: body mass index
HbA1c: glycosylated hemoglobin A1c
ICD-10: 10th revision of the World Health Organization’s International Classification of Disease ICPC = International Classification of Primary Care
LDL: low-density lipoprotein
sBP: systolic blood pressure
SD: standard deviation
T2D: type 2 diabetes
