# Supplementary material for: Recurrent prescription of sleep medication among primary care patients with type 2 diabetes: an observational study of real-world registry data
Source: BMC Prim Care. 2023 Apr 5;24:90. doi: 10.1186/s12875-023-02045-1 (PMC10074898; doi:10.1186/s12875-023-02045-1)
Supplement: Supplementary file 1 — Supplementary Material 1 [file 12875_2023_2045_MOESM1_ESM.docx]

| **Supplement 1. Sleep medication subcategories and their characteristics. Percentages and numbers presented, unless otherwise indicated.** | | | | | |
| --- | --- | --- | --- | --- | --- |
|  | **Recurrent sleep medication prescription** | | | **No recurrent sleep medication prescription** (n=3,242) |  |
|  | *Previous medication*  (=626) | *Ongoing medication*  (n=339) | *Subsequent medication*  (n=301) |  | P value |
| Age, mean (SD) | 72.8 (11.8) | 73.3 (11.1) | 74.8 (12.6) | 68.5 (11.7) | <0.001 |
| Sex |  |  |  |  | <0.001 |
| Females | 52.7 (330) | 55.8 (189) | 54.5 (164) | 42.6 (1,381) |  |
| Males | 47.3 (296) | 44.2 (150) | 45.5 (137) | 57.4 (1,861) |  |
| Sleep medication (percentage of ‘yes’ responses are presented) |  |  |  |  |  |
| Benzodiazepine-like medication | 61.5 (385) | 59.0 (200) | 44.9 (135) | 1.0 (33) | <0.001 |
| Melatonin | 38.0 (238) | 52.2 (177) | 48.8 (147) | 0.9 (28) | <0.001 |
| Mirtazapin | 32.1 (201) | 42.5 (144) | 35.9 (108) | 0.5 (17) | <0.001 |
| Temazepam | 18.8 (118) | 24.5 (83) | 14.0 (42) | 0.3 (11) | <0.001 |
| Nitrazepam | 0 (0) | 0.9 (3) | 0 (0) | 0 (0) |  |
| Doxylamine | 0.3 (2) | 1.2 (4) | 0 (0) | 0 (0) | <0.001 |
| Doxepin | 0 (0) | 0 (0) | 0 (0) | 0 (0) |  |
| Trimipramine | 4.8 (30) | 6.2 (21) | 5.6 (17) | 0.1 (4) | <0.001 |
| Trazodone | 0 (0) | 0 (0) | 0 (0) | 0 (0) |  |
| Number of sleep medications, median (IQR) | 11.0 (4.0-26.0) | 14.0 (5.5-31.5) | 6.0 (3.0-15.0) | NA | <0.001 |
| Sleep apnea |  |  |  |  | <0.001 |
| Yes | 24.6 (154) | 24.8 (84) | 19.3 (58) | 8.3 (268) |  |
| No | 75.4 (472) | 75.2 (255) | 80.7 (243) | 91.7 (2,974) |  |
| Non-apnea sleep disorder |  |  |  |  | <0.001 |
| Yes | 24.3 (152) | 22.4 (76) | 16.6 (50) | 8.3 (270) |  |
| No | 22.4 (76) | 77.6 (263) | 83.4 (251) | 91.7 (2,972) |  |
| Body mass index, mean (SD) | 29.8 (6.6) | 29.1 (5.6) | 28.3 (6.2) | 30.0 (5.8) | <0.001 |
| Missing, % (n) | n=73 | n=103 | n=55 | n=784 |  |
| Depression |  |  |  |  | <0.001 |
| Yes | 20.8 (130) | 22.7 (77) | 15.9 (48) | 7.2 (234) |  |
| No | 79.2 (496) | 77.3 (262) | 84.1 (253) | 92.8 (3,008) |  |
| Long-acting insulin |  |  |  |  | <0.001 |
| Yes | 33.5 (210) | 31.6 (107) | 29.6 (89) | 21.8 (707) |  |
| No | 66.5 (416) | 68.4 (232) | 70.4 (212) | 78.2 (2,535) |  |
| Antihypertensive medication of any kind |  |  |  |  | <0.001 |
| Yes | 87.1 (545) | 89.4 (303) | 78.4 (236) | 78.5 (2,544) |  |
| No | 12.9 (81) | 10.6 (36) | 21.6 (65) | 21,5 (698) |  |
| Lipid lowering medication of any kind |  |  |  |  | <0.001 |
| Yes | 74.0 (463) | 69.3 (235) | 53.8 (162) | 66.0 (2,140) |  |
| No | 26.0 (163) | 30.7 (104) | 46.2 (139) | 34.0 (1,102) |  |
| Achieved HbA1c target (<53 mmol/mol) |  |  |  |  | <0.001 |
| Yes | 64.4 (403) | 68.7 (233) | 68.1 (205) | 72.4 (2,348) |  |
| No | 35.6 (223) | 31.3 (106) | 31.9 (96) | 27.6 (894) |  |
| Achieved LDL target (<2.5 mmol/l) |  |  |  |  | <0.001 |
| Yes | 63.3 (396) | 59.3 (201) | 50.8 (153) | 53.2 (1,724) |  |
| No | 36.7 (230) | 40.7 (138) | 49.2 (148) | 46.8 (1,518) |  |
| Achieved systolic BP target (<135 mmHg) |  |  |  |  | 0.014 |
| Yes | 42.3 (223) | 46.6 (145) | 54.3 (146) | 47.0 (377) |  |
| No | 57.7 (304) | 53.4 (166) | 45.7 (123) | 53.0 (425) |  |
| *Missing* | n=99 | n=28 | n=32 | n=2,440 |  |
| HbA1c=glycosylated hemoglobin A1c  LDL=low-density lipoprotein  sBP=systolic blood pressure  SD=standard deviation  IQR=interquartile range | | | | | |
